# Association between body composition and khat chewing in Ethiopian adults

**DOI:** 10.1186/s13104-015-1601-2

**Published:** 2015-11-14

**Authors:** Tsinuel Girma, Andualem Mossie, Yesufe Getu

**Affiliations:** Department of Paediatrics and Child Health, College of Public Health and Medical Sciences, Jimma University, Jimma, Ethiopia; Department of Biomedical Sciences (Physiology), College of Public Health and Medical Sciences, Jimma University, Jimma, Ethiopia; Save the Children International, Jimma, Ethiopia

**Keywords:** Body composition, Khat chewing, Fat mass, Fat free mass, Bioimpedance, Catha edulis Forsk

## Abstract

**Background:**

Khat (Catha edulis Forsk) is a psychostimulant herb widely cultivated and used in Ethiopia. The link between khat use and body composition is little known.

**Objective:**

The aim was to determine the association between body composition and khat use.

**Methods:**

We recruited 415 individuals 18–78 years of age from Jimma Town. Sociodemographic and lifestyle data were collected using structured questionnaires. Fat mass (FM, kg) and fat-free mass (FFM, kg) were estimated from whole-body bio impedance. Weight (kg), FM and FFM were indexed to height (m) as BMI (kg/m^2^), fat mass index (FMI) (kg/m) and fat-free mass index (FFMI) (kg/m), respectively. Independent predictors of BMI, FMI and FFMI were identified using regression analysis.

**Result:**

Participants’ mean ± SD of age was 37 ± 13 years and 205 (53.2 %) were males. Mean ± SD of BMI, FMI and FFMI were 21.0 kg/m^2^ ± 4.1, 6.8 kg/m ± 5.2 and 27.9 kg/m ± 3.8, respectively. Multivariable model showed that, compared with non-chewers, khat chewers had a lower BMI (B = −1.56, 95 % CI −0.78, −2.33) and FMI (B = 2.19, 95 % CI −1.32, −3.06). FMI was lower in cigarette smokers than non-smokers by −1.36 kg/m (95 % CI −0.23, −2.49). Concurrently, khat and cigarette users increased FMI by 2.78 (95 % CI 0.11, 5.44). FFMI decreased with age (B = −0.02, 95 % CI −0.05, −0.002).

**Conclusion:**

FMI and BMI were lower in khat users than non-users, but there was no difference in lean mass. The consequence of this body composition change should be investigated.

## Background

The psychostimulant herbal plant, khat (*Catha edulis* Forsk) is produced abundantly in East Africa and the habit of chewing khat leaves among certain populations of Ethiopia dates back centuries [1]. Fresh khat leaves and young buds are usually chewed to obtain a state of euphoria [2]. Khat contains the active chemicals cathinone and cathine, which have a structural and functional resemblance to amphetamine and share many pharmacologic features in common [3]. Khat has various physiologic and metabolic effects associated with a decrease in appetite and body weight, possibly mediated via increasing the release of leptin from the stomach [4].

Khat chewing and its impact on elevated plasma leptin, esterified fatty acid production and resulting lipodystrophy, which leads to further problems on the cardiovascular system, urinary system, gastrointestinal tract, spermatogenesis, and impotence and loss of appetite have been summarised in previous studies [5, 6]. Previous studies used weight measurement to assess the effect of khat chewing on the body [6]. However, data on the effect of khat on the different constituents of body mass i.e. body composition are lacking.

Body composition is a term used to describe the different constituents that make up a human’s body weight. According to elemental, chemical, anatomical or fluid components, body composition investigation involves subdividing body weight into two or more compartments. The classic two-compartment model divides the body mass into fat mass (FM) and fat-free mass (FFM) compartments. The FM comprises of all lipids, and the FFM includes water, protein, and mineral components [7].

There are several different methods used to identify body composition, such as body mass index, skin folds and bioelectrical impedance analyses [8]. Bioelectrical Impedance Analysis (BIA) is an easy, cheap, valid and non-invasive method, in which resistance to a low electric current level with a frequency of 50 kHz is proportional to the percentage of FM [8]. The BIA technique considers a two-compartment model based on the principle that an electrical current flows more rapidly through tissues with higher water and electrolyte content than through fatty tissues, which are dehydrated.

Khat is produced in Jimma area in abundance and many people in the region use it for mood elevation. Based on simple observation, khat users are physically tiny and bony, probably because chemicals in khat may suppress their appetite. There are only limited data documented so far about the association between khat use and body composition. Moreover, the current study is the first of its type to determine body composition using BIA to measure FM and FFM among khat users and non-users. Therefore, the main aim of the present study was to determine the association between body composition and khat use by measuring the actual FM and FFM, helping fill the current gap in knowledge.

## Methods

The study was conducted in Jimma town, Ethiopia, from February 1 to March 30, 2012. The total projected population of the town from 2007 central statistical agency (CSA) census report was 120, 960 [9]. Individuals aged 18 and above living in Jimma town were used as the source population. Pregnant women or lactating mothers with babies under 6 months, physical disability, clients with history of chronic illness such as HIV/AIDS and diabetes mellitus were excluded.

The sample size was calculated using single population proportion formula, assuming a confidence level of 95 and 10 % allowance for non-response rate. The proportion of overweight (P) plus obesity, which is 43.3 % (Over weight = 24.1 % and obesity = 19.2 %) taken from studies done in Tanzania [10].

Multistage simple random sampling technique was employed to select the study participants. Six kebeles (the smallest administrative unit of Ethiopia) were selected from a total of 13 kebeles found in the town by a lottery method. The potential sample size was allocated to each study kebele by population proportion. Urban health extension workers (UHEW) invited adults from their respective kebeles to participate in the study. Participants who fulfilled the inclusion criteria were selected for interview, anthropometric and body composition measurement.

The data collection was done at six sites, i.e. in the office of urban health extension workers in each study kebeles. Five well trained nurses were involved in the data collection activities under strict supervision by the investigators.

Data on sociodemographic and lifestyle habits such as khat chewing, alcohol drinking, cigarette smoking and level of physical activity were obtained from a pre-tested interviewer-administered structured questionnaire. The WHO stepwise approach for chronic disease risk factor surveillance in developing countries was used with certain modifications [11].

Physical activity level was assessed based on self-reported physical commotion of participants for the period of work. “Physically active” was defined as a person doing activities that require hard physical effort and cause large increases in breathing or heart rate (like carrying or lifting heavy loads, digging or construction work) for at least 10 min continuously within a day such as crop harvesting, fishing and hunting. “Physically inactive” was defined as a person doing activities that require smaller physical effort and causes small increases in breathing or heart rate like brisk walking, carrying light loads. “Current khat chewer” was defined as khat chewing at least once in the past 30 days.

Weight was measured with subjects minimally dressed and barefoot (TANITA, BC-418MA model) and recorded at a precision of 0.1 kg. Height was measured using a height measurement board of clients in upright position with barefoot at a precision of 0.1 cm [12]. Waist circumference was measured in triplicates using non-elastic measuring tape midway between the inferior margin of the last rib and the iliac crest at the end of expiration [13] and the mean was calculated for analysis. The tape was first calibrated by a ruler.

Whole-body bioimpedance was measured using bioimpedance analyser (TANITA Corporation, Tokyo, Japan), and according to a protocol by the manufacturer [14]. Age, sex and height of the individuals were fed into the analyser and estimates of FFM (kg), FM (kg) and body mass index (BMI, kg/m^2^) generated by the machine were recorded. FM and FFM were indexed for height as FMI and FFMI during analysis. Body fat percentage >25 % for males and >33 % for females were used as a cut-point for excess body fat (EBF) [15].

Data was double entered into EpiData (Odense, Denmark) and analyzed using Stata 12 (StataCorp LP, College station, Texas, USA). The mean differences in continuous outcomes between groups were tested using the student’s *t* test with p ≤ 0.05 considered to be significant. Hierarchical multiple linear regression analysis was employed to find independent predictors of the outcome variable. The following covariates were included in the regression model: Age, sex, ethnicity, religion, marital status, educational status, monthly income, cigarette smoking, alcohol drinking, and physical activity. We used interaction term in the model to test if gender, cigarette smoking, alcohol drinking moderated the link between khat use and body composition measures.

Ethical clearance for the study was obtained from an Ethical Review Board of Jimma University. Client’s written consent was taken and confidentiality was maintained. For those illiterates, we took their fingerprint as a signature.

## Results

We studied 415 participants. All data from 30 (7.2 %) of them were excluded because of incompleteness. The mean ± SD of age of the participants was 37 ± 13 years and 205 (53.2 %) of them were males (Table 1). Among the participants, 226 (58.7 %) and 186 (48.3 %) were from the Oromo ethnic group and Muslims, respectively.

**Table 1:** Selected sociodemographic characteristics of the study participants (N = 385)

Sociodemographic variables	Categories	N (%)
Age, year	mean ± SD	37 ± 13
Sex	Male	205 (53.2)
Ethnicity	Oromo	226 (58.7)
	Amhara	63 (16.4)
	Gurage	27 (7.0)
	Dawuro	23 (6.0)
	Tigrie	18 (4.7)
	Others^a^	28 (7.3)
Religion	Muslim	186 (48.3)
	Orthodox	148 (38.4)
	Protestant	44 (11.4)
	Catholic	7 (1.8)
Marital status	Married	263 (68.3)
	Single	72 (18.7)
	Divorced	37 (9.6)
	Widowed	13 (3.4)
Educational status	Illiterates	40 (10.4)
	Primary school	164 (42.6)
	Secondary school	96 (24.9)
	Tertiary	85 (22.1)
Family monthly income, Birr	<1000	85 (22.1)
	1000–2000	138 (35.8)
	>2000	162 (42.1)

19.31 Birr = 1USD

^a^Kefficho, Yem, Selte

The proportions of those with history of ever chewing Khat, ever smoking cigarettes and ever drinking alcohol were 186 (48.3 %), 60 (15.3 %) and 185 (48.1 %), respectively (Table 2).

**Table 2:** Substance use profile of 385 Ethiopian adults

	n (%)
Ever chew khat	186 (48.3)
Current khat chewing	138 (35.8)
Ever smoke cigarette	60 (15.3)
Current cigarette smoking	43 (11.2)
Ever drink alcohol	185 (48.1)
Current alcohol drinking	167 (43.4)

Compared with non-khat chewers, chewers had a lower waist circumference [Diff = −3.6 cm, 95 % CI ([−5.8,–1.4]), df. = 383 (t = 12.0, p < 0.001), BMI [Diff = −5.9 kg/m^2^, 95 % CI ([−8.2, −3.6])], df. = 383 (t = 7.6, p < 0.001), fat mass [Diff = −7.9 kg, 95 %CI ([−9.4, −6.4])], df. = 383(t = 12.0, p < 0.001) and trunk fat mass [Diff = −3.8 kg, 95 % CI ([−4.6, −3.0])], df. = 383, (t = −6.28, p < 0.001) of (Table 3). Whereas FFMI of khat chewers was higher than that of the non-chewers, [Diff = 1.7 kg/m, 95 % CI ([1.0, 2.4])], df. = 383, (t = 4.56, p < 0.001).

**Table 3:** Association of body composition estimated from whole-body bioimpedance and khat chewing in 385 Ethiopian adults

	Current khat chewing^a^		Diff [95 % CI]	p-value
	Yes (n = 138)	No (n = 247)		
Weight (kg)	55.8 (10.1)	59.5 (12.0)	−3.6 [−5.8, −1.4]	<0.001
Waist circumference (cm)	77.7 (10.2)	83.6 (12.4)	−5.9 [−8.2, −3.6]	<0.001
Body mass index (kg/m^2^)	19.7 (3.0)	22.3 (4.3)	−2.6 [−3.4, −1.9]	<0.001
Fat mass (kg)	7.1 (5.8)	15.0 (8.6)	−7.9 [−9.4, −6.4]	<0.001
Fat mass index (kg/m)	4.3 (3.4)	9.2 (5.3)	−5.0 [−5.9, −4.1]	<0.001
Fat free mass (kg)	48.7 (8.2)	44.4 (7.1)	4.3 [2.7, 5.8]	<0.001
Fat free mass index (kg/m)	28.8 (3.9)	27.1 (3.4)	1.7 [1.0, 2.4]	<0.001
Trunk fat mass (kg)	3.5 (3.2)	7.3 (4.8)	−3.8 [−4.6, −3.0]	<0.001

^a^Values are given as mean ± standard deviation and difference between groups was tested with independent sample t-test

As shown in Table 4, an inverse relationship was observed between the frequency of khat chewing and the percentage of individuals having EBF (p = 0.005). Similarly, the proportion of individuals with EBF was lower among those who chewed khat for two or more years compared with chewing for shorter duration (8.1 vs. 20.3 %, p = 0.04).

**Table 4:** Association of the frequency and duration of khat chewing with excess body fat estimated from whole-body bioimpedance among 385 Ethiopian adults

	Excess body fat (n %)^a^		χ^2^	P-value
	Yes	No		
Khat chewing status				
None chewers	73 (36.7)	126 (63.3)	13.3	0.01
Ex-chewers	15 (29.2)	34 (70.8)		
Current chewers	25 (18.2)	118 (81.8)		
Frequency of chewing				
Every day	4 (12.1)	29 (87.9)	12.6	0.005
2–3 days/week	5 (12.5)	35 (87.5)		
Once a week	5 (12.8)	34 (87.2)		
Occasionally	11 (43.3)	15 (57.7)		
Duration of chewing				
<2 year	13 (20.3)	51 (79.7)	4.3	0.04
≥2 year	6 (8.1)	68 (91.9)		

^a^Body fat was; excess body fat = body fat percentage >25.0 % for males and >33.0 % for females

In the multiple regression model (Table 5), BMI increased with age (Beta, B) = 0.03, 95 % CI 0.00, 0.057) and monthly income (B = 0.002, 95 % CI 0.001, 0.003). Similarly FMI increased with age (B = 0.06, 95 % CI 0.03, 0.09), monthly income (β = 0.002, 95 % CI 0.002, 0.003), with lesser physical activity level (B = 0.94, 95 % CI 0.09, 1.80) and was higher in females than males by 3.12 (95 % CI 2.25, 3.99). On the other hand, khat chewers had a lower BMI (B = −1.56, 95 % CI −0.78, −2.33). The interaction term discovered that using both khat and cigarette increased FMI by 2.78 (95 % CI 0.11, 5.44). FFMI decreased with age (B = −0.02, 95 % CI −0.05, −0.002) and was lower in females than males by −4.66 (95 % CI −5.26, −4.05).

**Table 5:** Predictors of body composition estimated from whole-body bioimpedance among 385 Ethiopian adults

	BMI (kg/m^2^)	p-value	FMI (kg/m)	p-value	FFMI (kg/m)	p-value
	B (95 % CI)		B (95 % CI)		B (95 % CI)	
Age, year	0.03 (0.00, 0.057)	0.031*	0.06 (0.03, 0.09)	<0.001*	−0.02 (−0.05, −0.002)	0.036*
Sex, female	0.52 (−0.24, 1.28)	0.180	3.12 (2.25, 3.99)	<0.001*	−4.66 (−5.26, −4.05)	<0.001*
Monthly income, Birr	0.002 (0.001, 0.003)	<0.001*	0.002 (0.002, 0.003)	<0.001*	0.001 (0.001, 0.0013)	<0.001*
Current khat chewers**	−1.56 (−0.78, −2.33)	<0.001*	−2.19 (−1.32, −3.06)	<0.001*	0.09 (−0.66, 0.82)	0.817
Cigarette smokers	0.76 (−0.25, 1.78)	0.141	−1.36 (−0.23, −2.49)	0.032*	0.40 (−0.49, 1.29)	0.379
Current khat chewer^##,^ cigarette smoker	2.07 (−0.34, 4.48)	0.09	2.78 (0.11, 5.44)	0.041*	0.09 (−2.04, 2.22)	0.936
Physical activity level, inactive	0.60 (−0.16, 1.37)	0.123	0.94 (0.09, 1.80)	0.021*	0.20 (−0.48, 0.87)	0.569

All dependent variables were indexed to height (m) as *BMI* body mass index; *FMI* fat mass index and *FFMI* fat free mass index; *19.31 Birr* 1 USD, *B* regression coefficient. Age, sex, ethnicity, religion, marital status, educational status, monthly income, cigarette smoking, alcohol drinking and physical activity were included in the model

* Significant

** Khat chewing at least once in the past 30 days

## Concurrent of khat chewing and smoking cigarette

## Discussion

Our study revealed major findings, i.e. khat chewers were lighter and less-fatter than non-chewers whereas no relationship was found between khat chewing and FFMI. Moreover, older individuals and females, compared with males, were fatter and had a lesser lean body mass. Cigarette smoking was negatively related with FMI.

A decrease in FMI and BMI among khat chewers may be due to a decrease in food intake as a result of appetite suppressant effects of khat [16]. Khat contains active ingredients called cathinone and cathine, which are amphetamine-like compounds that can inhibit the appetite centre in the hypothalamus [17]. These effects are probably mediated via increasing the level of leptin in plasma and have fat mobilization effect by acting indirectly. Apart from its central effect, cathinone enhances sympathomimetic activity leading to a delay in gastric emptying [18].

On the other hand, individuals who use both khat and cigarette had higher fat mass. This may be related to the potentiating effect of Khat chewing on cigarette smoking. That means khat chewer smokers smoke more cigarette than non-chewer smokers. Therefore, adults who consume both khat and cigarette would have greater smoking profile and have a greater body weight than do light smoker [19].

FMI increased while the FFMI decreased with age, which is in line with study results done in Bulgaria which showed a positive association between age and FM but a decrease in FFM [20]. With age, metabolism slows because of hormonal and enzymatic factors and physical activity decreases [21], which in turn may decrease energy expenditure and result in the accumulation of body fat.

Another important result of the present study was higher FMI among individuals with relatively higher income, this agrees with findings in Nigeria [22]. Individuals with higher income are less likely to be engaged in labour demanding physical activities and had a sedentary lifestyle, thus expending less energy. In addition, adults with higher income have a chance to get more and higher calorie diet than low income adults.

Generalization of the finding in this study will be limited as all the participants were recruited from an urban setting. Whilst our findings are still useful to offer comparative analysis within the study population, validated equations would help to compare findings at the global level.

In conclusion, this study demonstrated that older individuals were not only heavier and fatter, but also had a lesser lean mass than young respondents. Despite comparable weight with males, females were fatter and had lesser lean mass. Both khat users and cigarette smokers had less body fat compared with their respective non-users. But, individuals who use both khat and cigarette had higher fat mass. However, khat use did not affect lean mass. Body fat decreased with an increase in the level of physical activity, though no effect was found on lean mass.

Animal studies are required to confirm the effect of khat on body composition and to define its mechanism. Additionally, it would be valuable to determine the socioeconomic as well as the health consequences of body composition variation due to khat use.
